# Loneliness, emotional support and the mental health of young adults and their parents in New York, US during the COVID-19 pandemic: a cohort study

**DOI:** 10.1186/s12888-024-06305-x

**Published:** 2024-11-25

**Authors:** Abosede Akinkuowo, Keely Cheslack-Postava, Norbert Skokauskas, Christina W. Hoven

**Affiliations:** 1 Global Health, Department of Public Health and Nursing, Faculty of Medicine and Health Sciences, Norwegian University of Science and Technology (NTNU), Trondheim, Norway; 2 Department of Psychiatry, Columbia University, New York State Psychiatric Institute, New York, NY USA; 3 Department of Mental Health, Regional Centre for Child and Youth Mental Health and Child Welfare (RKBU Central Norway), Norwegian University of Science and Technology (NTNU), Trondheim, Norway; 4 Epidemiology and Psychiatry, Columbia University-New York State Psychiatric Institute, New York, NY USA

**Keywords:** COVID-19, Depression, Emotional support, Loneliness, Substance use, New York City

## Abstract

**Background:**

The coronavirus disease (COVID-19) pandemic led to social isolation and widespread lockdown, resulting in loneliness and lack of emotional support, which have been associated with adverse mental health outcomes. This study aims to explore the relationship of loneliness and emotional support with depression and substance use among young adults and their parents during the COVID-19 pandemic.

**Methods:**

A cohort of 1227 participants was recruited from three ongoing cohort studies in the metropolitan area of New York City, USA. Data was collected through telephone interviews using preset questionnaires during wave 1 (March-August 2020), wave 2 (September 2020-February 2021) and wave 3 (March-August 2021) of the COVID-19 pandemic. Logistic regression models were used to test the association between loneliness and emotional support, and the mental health outcomes; depression and substance use, with adjustments made for age, gender, race, employment status, living conditions, and marital status. Additionally, the effect modification of respondent type and living alone or with others was examined.

**Results:**

At wave 1, loneliness showed a significant positive association with depressive symptoms (OR: 2.56, 95%CI: 2.19-3.00, P = < 0.001) and an increase in substance use, such as smoking tobacco using cigarettes, pipes, or cigar (OR: 1.59, 95%CI: 1.24–2.04, P = < 0.001), alcohol consumption (OR: 1.23, 95%CI: 1.07–1.42, *P* = 0.003), and marijuana/other substances (OR: 1.57, 95%CI: 1.26–1.96, P = < 0.001). Conversely, emotional support showed a significant negative association with depressive symptoms (OR: 0.71, 95%CI: 0.62–0.81, P = < 0.001) but a non-significant association with increase in tobacco smoking using vapes and e-cigarettes, alcohol consumption and marijuana and other substances. However, a significant negative association was observed between emotional support, and increased tobacco smoking using cigarettes, pipes, or cigar (OR: 0.73, 95%CI: 0.58–0.93, *P* = 0.011). The associations of loneliness and emotional support with mental health outcomes were stronger at wave 2 compared to wave 1. The modifier effects of respondent type and living condition were non-significant.

**Conclusions:**

This study indicates that increased loneliness is associated with a higher likelihood of depression and substance use, while higher emotional support is linked to a reduced likelihood of depressive symptoms during the COVID-19 pandemic.

## Background

The Coronavirus disease (COVID-19) pandemic was accompanied by social isolation and widespread lockdown. According to the World Health Organization (WHO), these events have been associated with poor mental health outcomes globally [1]. New York City is the most populous city in the United States and was largely affected by the COVID-19 pandemic. New York City recorded 17,750 deaths by the 31st of May 2020 and residents were confined at home for 78 days [2]. Lockdowns and social isolation may lead to social outcomes like unemployment and increased violence, as well as mental health-related outcomes including increased substance abuse and higher numbers of suicides [3–5]. Loneliness and lack of emotional support have been identified to be commonplace in such a case of pandemic with mass confinement [6]. They result in psychological outcomes like stress, depression, elevated stress levels, post-traumatic stress disorder, substance abuse and suicide [6].

Previous studies have associated loneliness with mental health conditions like depression, anxiety disorders, suicidal behaviors and death [7, 8]. Loneliness has also been associated with relapse in those with drug addictions, usually due to the fear, anxiety, irritability and boredom that come with loneliness [9]. On the other hand, studies have reported that strong and adequate emotional and social support from family, friends or co-workers are related to positive mental health outcomes, especially during stressful events [10, 11]. However, a body of literature has shown that the COVID-19 pandemic may have increased the difficulty of utilizing emotional support as a coping strategy [11]. This is because of the social isolation and confinement that characterized the pandemic and made it difficult for social and emotional connection among people [11].

Despite existing knowledge about the association of loneliness and emotional support on mental health outcomes, there is still a knowledge gap on how the COVID-19 pandemic impacted these relationships, especially in young adults living in a country with long days of confinement during the pandemic.

Considering the effects of loneliness and emotional support on mental health outcomes especially during a pandemic such as COVID-19, may help identify areas of focus for interventions during future pandemics to improve mental health outcomes. This study was conducted to determine the association of loneliness and emotional support with the prevalence of depression and substance use behaviors in young adults and their parents, who were recruited from metropolitan New York City, based on participation in ongoing cohort studies.

## Methods

### Study participants

This study was an observational cohort of young adults and their parents who were participants in three ongoing cohort studies based in the New York City metropolitan area. A longitudinal telephone survey was conducted over 3 waves during the COVID-19 pandemic (Wave 1: March-August 2020, Wave 2: September 2020-February 2021, Wave 3: March-August 2021). Each cohort was composed of young adults and parents of young adults. Only data from wave 1 and wave 2 were included in this study because information on the variables of interest were collected in those waves.

**Fig. 1:**
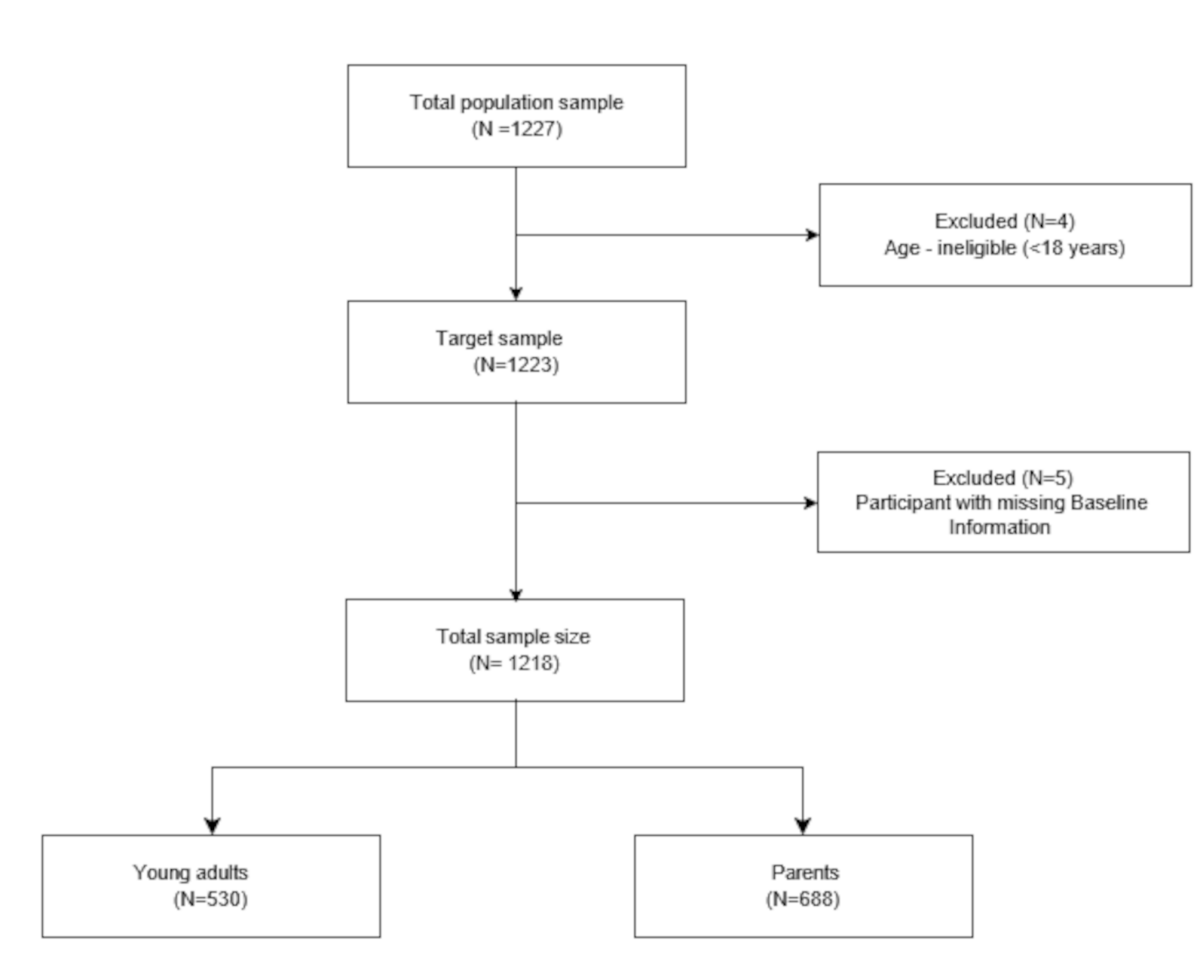
Flowchart of the study sample size

This study recruited a total of 1227 participants (1222 at wave 1, 927 at wave 2, and 815 at wave 3; with a mean of 2.4 observations per person). Of the 1227 participants that were recruited into the study, 9 participants were excluded from the data analysis. Five of the excluded participants had missing wave 1 information while the remainder were below 18 years of age. Therefore, 1218 participants were included in the data analysis. Figure 1 shows the flowchart of the study sample size.

Flowchart of the study sample size for the analysis of the association between loneliness and emotional support and depression or increased substance among adults in New York City.

### Data collection

Data was collected through a telephone survey using pre-set questionnaires. The survey was administered by trained telephone interviewers, who were blinded to the original study population of participants. Interviews were conducted in the language chosen by the participant (English, 91%, Spanish, 8% and Mandarin, 1%), and the data was entered by the interviewer directly into a password-protected database. The current study was based on a secondary analysis of questions from the survey as described below.

### Measurement tools

The exposure variables are loneliness and emotional support, and the outcome variables are depression and increase in substance use.

### Loneliness scores

To measure the level of loneliness in both waves 1 and 2, participants were asked whether they feel alone and apart from others, feel left out, feel that they are no longer close to anyone, feel alone and feel lonely (see Appendix A in [12]). Each question was categorized according to response options; Never, Rarely, Sometimes, Usually and Always. Before analysis, these categories were assigned numerical values of 1–5 respectively. The mean of the numerical values of the responses of participants to the five loneliness-related questions was taken for each participant as the loneliness score.

### Emotional support score

The emotional support score was used to rate the level of emotional support an individual experienced. The emotional support scores were obtained using the National Institutes of Health (NIH) Toolbox Emotional Support score in both wave 1 and 2 studies [13]. The scale consists of 8 items, such as questions including whether there are people the individual can talk to if they are upset, have someone who will listen to them when they need to talk, have someone who understands their problems, among other questions (see Appendix A in [12]). Each question was categorized according to the response options; Never, Rarely, Sometimes, Usually and Always. The total score ranges from 8 to 40, with higher values indicating greater level of emotional support [13].

### Depressive symptoms severity

Participants in both waves 1 and 2 were screened using the Patient Health Questionnaire (PHQ-8) [14]. The PHQ-8 consists of 8 items, each scored 0–3 (see Appendix A in [14]). High scores signifies high depressive symptom severity [14]. Participants were categorized into two groups based on whether they have experienced the outcome, if they meet the cutoff point for moderate to severe depressive symptoms (10–24) or not (0–9) [14].

### Substance use

Substance use was measured based on participants’ response to self-reported questionnaire in both waves 1 and 2, with responses categorized as increase in: smoking tobacco using cigarettes, pipes or cigar; smoking tobacco using vapes or e- cigarettes; alcohol drinking; and marijuana use and other drugs. Participants’ response of ‘yes’ that they had increased their use of these substances in the last 30 days before the survey was considered positive for each outcome. The questions were developed for this survey and consisted of yes or no response options to the question of whether participants had increased their use of each substance in the past 30 days.

### Covariates

Age, gender, race, employment status, condition of living and marital status were studied as covariates and potential confounders. Age was included as self-reported age (in years) of participants at wave 1. Gender was categorized based on participants’ response on whether they were female or male at birth. Race was included as participants’ self-reported race. Participants response of ‘yes’ that they were employed at the time of the interview or ‘no’ that they were not, were used to measure employment status. Condition of living was measured as whether the participants reported that they were living alone or with at least one other person. Participants reported whether they were married and living with spouse, living with a partner as though married, separated and not living with someone, divorced and not living with someone, widowed and not living with someone, or never married and not living with someone. These responses were used to measure marital status.

### Statistical analysis

First, descriptive statistics were used to summarize the data. Loneliness scores and emotional support scores were standardized, and logistic regression models were used to examine the association between loneliness and emotional support with depression and with increase in substance use. Covariates including age, gender, race, employment status, condition of living and marital status were considered as potential confounders and were also adjusted for in the analysis. Similar analysis was done for both wave 1 and wave 2, with separate logistic regression models examining each exposure-outcome association cross-sectionally at wave 1 and wave 2. The potential effect modification of living condition and respondent types were examined on the associations between the exposure variables and the outcome variables in the wave 1 analysis. The effect modification was tested by adding product terms to logistic regression models. The interactions of potential effect modifiers with the exposures were considered significant when p-values of regression coefficients, β for the product terms were less than 0.05. In addition, loneliness and emotional support were simultaneously included in the same adjusted models to examine their independent associations. A sensitivity analysis was conducted to examine the prospective associations between wave 1 loneliness or emotional support and wave 2 depression or increased substance use.

Statistical significance was set at a p value of < 0.05.

The R programming tool version 4.2.2 for data management, analysis and visualization was used in this study.

## Results

### Participant wave 1 characteristics

The mean age of the 1218 participants included in the analysis was 42.7 years. Of these participants, 530 (43.5%) were young adults with a mean age of 24.5 years and 688 (56.5%) were parents of young adults with a mean age of 57.3. Other information at wave 1 is presented in Table 1.

**Table 1:** Participants’ characteristics at wave 1, *N* = 1218

Categories	Frequency	Relative frequency (%)
**Gender**		
Male	448	36.78
Female	770	63.22
**Respondent**		
Young adults	530	43.51
Parents	688	56.49
**Race** ^#^		
American Indian/ Eskimo	4	0.33
Asian	73	6.07
Native Hawaiian/Pacific islander	2	0.17
Black/ African American	87	7.23
White	555	46.13
More than 1 race	44	3.66
Other/unknown	90	7.48
Hispanic	348	28.93
Age*		
18–25	317	26.07
26–35	200	16.45
36–50	167	13.73
51–65	452	37.17
66 and above	80	6.58
**Marital Status** ^β^		
Married, living with someone	526	43.22
Living with a partner as though married	51	4.19
Separated, and not living with someone	43	3.53
Divorced, and not living with someone	84	6.90
Widowed, and not living with someone	20	1.64
Never married, and not living with someone	420	34.51
Others	73	6.00
**Condition of living** ^π^		
Live with at least one other person	1120	92.03
Live alone	97	7.97
**Employment**		
No	454	37.27
Yes	764	62.73
**Personal Income** ^Ω^		
Less than $1000	165	14.16
$1,000–4,999	11	0.94
$5,000–9,999	95	8.15
$10,000–19,999	111	9.53
$20,000–34,999	138	11.85
$35,000–54,999	171	14.68
$55,000–74,999	145	12.45
$75,000–99,999	123	10.56
$100,000–199,999	159	13.65
$200,000 or more	47	4.03

**#=** 15 missing values, *=2 missing values, β = 1 missing value, π = 1 missing value, Ω = 53 missing values

### Loneliness and emotional support

At wave 1, the mean loneliness score for the participants was 2.083 (SD = 0.92, minimum = 1.0 and maximum = 5.0), and the mean emotional support score obtained by the participants was 34.59 (SD = 6.23, minimum = 8 and maximum = 40). At wave 2, the mean loneliness score that was obtained for participants was 1.96 (SD = 0.91, minimum = 1.0 and maximum = 5.0), and the mean emotional score obtained by the participants was 35.51 (SD = 5.72, minimum = 8 and maximum = 40). The frequencies of the participants’ responses to the loneliness questions at waves 1 and 2 are summarized in Table 2.

**Table 2:** Participants’ responses to loneliness parameters

	Wave 1						Wave 2						
**Loneliness Parameters**	Always	Usually	Sometimes	Rarely	Never	**Total**	Always	Usually	Sometimes	Rarely	Never	**Total**	
Feel alone and apart from others	6.9%	12.2%	37.3%	19.9%	23.7%	**100% ***n* = 1218	4.0%	8.5%	31.2%	23.6%	32.6%	**100%** *n* = 925	
Feel left out	2.7%	5.4%	21.9%	21.6%	48.3%	**100%** *n* = 1217	2.4%	4.1%	22.6%	22.2%	48.8%	**100% ***n* = 925	
Feel that you are no longer close to anyone	2.5%	3.4%	19.5%	18.9%	55.7%	**100%** *n* = 1216	2.8%	2.1%	19.8%	18.8%	56.4%	**100% ***n* = 924	
Feel alone	3.5%	5.3%	25.5%	18.3%	47.4%	**100%** *n* = 1218	3.1%	5.1%	21.1%	20.4%	50.3%	**100%** *n* = 925	
Feel lonely	3.4%	6.1%	30.2%	18.9%	41.4%	**100%** *n* = 1218	2.6%	5.1%	24.9%	21.7%	45.7%	**100%** *n* = 925	

*In both waves, the majority of the participants never experienced most of the loneliness parameters and only the minority reported to have always experienced most of the loneliness parameters

### Depression and substance use

At wave 1, the mean PHQ-8 raw score for all the participants was 5.42 (minimum = 0 and maximum = 24). Participants who experienced moderate to severe depressive symptoms were 221 (18.2%) while participants who did not were 991 (81.8%). At wave 2, only 925 participants participated. The mean PHQ-8 raw score for all the participants is 3.99 (minimum = 0 and maximum = 24). Participants who experienced moderate to severe depressive symptoms were 111 (12.0%) while participants who did not were 814 (88.0%). Details of the classification of participants’ level of depressive symptoms at both waves 1 and 2 are summarized in Table 3.

**Table 3:** Levels of depressive symptoms in participants

	Wave 1		Wave 2	
**Depression levels**	Frequency	Percentage	Frequency	Percentage
None - minimal	646	53.30%	612	66.16%
Mild	345	28.47%	202	21.84%
Moderate	144	11.88%	74	8.00%
Moderately severe	58	4.79%	26	2.81%
Severe	19	1.57%	11	1.19%
**Total**	1212	100%	925	100%

The majority of the participants experienced none to minimal depressive symptoms (wave 1; *n* = 646 and wave 2; *n* = 612) while only the minority experienced severe depressive symptoms (wave 1; *n* = 19 and wave 2; *n* = 11).

In the wave 1 study, 129 (10.6%) of the participants smoke tobacco using cigarettes, pipes or cigar, 59 (4.8%) smoke tobacco using vapes or e-cigarettes, 826 (67.8%) drink alcohol and 232 (19.1%) use marijuana or other drugs.

Participants reported whether they have increased their substance use in the past 30 days. Increase in alcohol consumption was the highest reported. Out of the 1218 participants in wave1, 226 (18.6%) reported increased alcohol consumption. Similarly, 44 (4.76%) increased alcohol consumption among the 925 participants in wave 2.

Details of participants’ increase in substance use at both waves 1and 2 are summarized in Table 4.

**Table 4:** Participants’ response to increase in substance use parameters

	Wave 1 (*N* = 1218)				Wave 2 (*N* = 925)			
**Increased substance use** **(past 30 days)**	Yes	(%)	No	(%)	Yes	(%)	No	(%)
Increased smoking of tobacco using cigarettes, pipes or cigar	50	4.11%	1168	95.9%	24	2.59%	901	97.4%
Increased smoking of tobacco using vapes or e-cigarettes	13	1.07%	1205	98.9%	9	0.97%	916	99.0%
Increased drinking alcohol	226	18.6%	992	81.4%	44	4.76%	881	95.2%
Increased use of marijuana or other drugs	65	5.34%	1153	94.7%	*	*	*	*

*Data for increased use of marijuana and other drugs was not available for wave 2

In both waves, alcohol was the substance for which use was most likely to have increased

### Association between Loneliness and the occurrence of depression and increased substance use

Results from the logistic regression analysis models of loneliness and each of depression and increased substance use at wave 1 showed that there was a significant positive association between loneliness and depression (OR: 2.56, 95%CI: 2.19-3.00, *P* < 0.001), and loneliness and these increased substance use parameters; smoking tobacco using cigarettes, pipes, or cigar (OR: 1.59, 95%CI: 1.24–2.04, *P* < 0.001), drinking alcohol (OR: 1.23, 95%CI: 1.07–1.42, *P* = 0.003), marijuana and other substances (OR: 1.57, 95%CI: 1.26–1.96, *P* < 0.001). After adjusting for potential confounders including age, gender, race, employment status, condition of living and marital status, these associations were of similar magnitude and remained statistically significant *(*Table 5*)*. However, the association between loneliness and increase in smoking tobacco using vapes or e-cigarettes which was not significant in the unadjusted model (OR: 1.56, 95%CI: 0.97–2.57, *P* = 0.067), showed a significantly positive association after adjusting for potential confounders (OR: 1.75, 95%CI: 1.02-3.00, *P* = 0.042) *(*Table 5*)*. The result from the models at wave 2 showed that there is a significant positive association between loneliness and depression (OR: 2.30, 95%CI: 1.90–2.79, *P* < 0.001). The association remained strong and statistically significant after adjusting for potential confounders (OR: 2.95, 95%CI: 2.64–3.31, *P* < 0.001). However, the associations between loneliness and increased substance use parameters were non-significant in this wave, except for the association between loneliness and increase in smoking tobacco using cigarettes, pipes or cigar, which became significant after adjusting for potential confounders (OR: 1.48, 95%CI: 1.22–1.79, *P* < 0.001). Details are shown in Table 5.

### Association between emotional support and the occurrence of depression and increased substance use

Results from the logistic regression analysis models of emotional support with each of depression and the increased substance use parameters at wave 1 showed a significant negative association between emotional support and depression (OR: 0.71, 95%CI: 0.62–0.81, *P* < 0.001). The associations were not meaningfully different after adjusting for potential confounders (OR: 0.73, 95%CI: 0.64–0.84, *P* < 0.001). On the other hand, the association between emotional support and increased substance use parameters were non-significant both in the unadjusted models and after adjusting for age, gender, race, employment status, condition of living and marital status as shown on Table 5. However, there was a significant negative association observed between emotional support and increase in smoking of tobacco using cigarettes, pipes, or cigar (OR: 0.73, 95%CI: 0.58–0.93, *P* = 0.011) which was similar after adjusting for potential confounders (OR: 0.76, 95%CI: 0.60–0.98, *P* = 0.034).

At wave 2, the models showed that the association between emotional support and depression was significantly negative (OR: 0.67, 95%CI: 0.56–0.79, *P* < 0.001) and the association was similar in the adjusted model (OR: 0.69, 95%CI: 0.58–0.82, *P* < 0.001). The relationship between emotional support and increased substance use parameters were not significant in either unadjusted or adjusted models, except for a significant positive association observed in the adjusted model with smoking of tobacco using vapes and e-cigarettes (OR: 2.32, 95%CI: 1.35–3.98, *P* = 0.002). Details are shown in Table 5.

**Table 5:** Association of loneliness and of emotional support with depression and increased substance use (waves 1 and 2)

		Unadjusted OR (95%CI)					Adjusted OR (95%CI) *				
		**Depression**	**Increase in Substance Use**				**Depression**	**Increase in Substance Use**			
			Smoking tobacco using cigarettes, pipes, or cigar	Smoking tobacco using vapes or e-cigarettes	Drinking alcohol	Marijuana and other substances		Smoking tobacco using cigarettes, pipes, or cigar	Smoking tobacco using vapes or e-cigarettes	Drinking alcohol	Marijuana and other substances
Wave 1	**Loneliness** ^**a**^	2.56 (2.19, 3.00) *P* < 0.001	1.59 (1.24, 2.04) *p* < 0.001	1.56 (0.97, 2.50) *p* = 0.07	1.23 (1.07, 1.42) *P* < 0.01	1.57 (1.26, 1.96) *p* < 0.001	2.49 (2.12, 2.93) *p* < 0.001	1.52 (1.18, 1.96) *P* < 0.01	1.75 (1.02, 3.00) *p* = 0.04	1.27 (1.09, 1.46) *P* < 0.01	1.54 (1.21, 1.97) *p* < 0.001
	**Emotional Support** ^**a**^	0.71 (0.62, 0.81) *p* < 0.001	0.73 (0.58, 0.93) *p* = 0.01	1.07 (0.60, 1.88) *p* = 0.83	1.13 (0.97, 1.32) *p* = 0.12	0.80 (0.64, 1.00) *p* = 0.05	0.73 (0.64, 0.84) *p* < 0.001	0.76 (0.60, 0.98) *p* = 0.03	1.09 (0.60, 1.98) *p* = 0.78	1.10 (0.94, 1.29) *p* = 0.22	0.89 (0.70, 1.13) *p* = 0.34
Wave 2	**Loneliness** ^**a**^	2.30 (1.90, 2.79) *p* < 0.001	1.08 (0.73, 1.59) *p* = 0.72	1.59 (0.90, 2.79) *p* = 0.11	1.21 (0.91, 1.60) *p* = 0.19	x	2.95 (2.64, 3.31) *p* < 0.001	1.48 (1.22, 1.79) *p* < 0.001	0.79 (0.55, 1.12) *p* = 0.19	1.01 (0.87, 1.18) *p* = 0.89	x
	**Emotional Support** ^**a**^	0.67 (0.56, 0.79) *p* < 0.001	0.84 (0.59, 1.19) *p* = 0.32	2.2 (0.69, 6.98) *p* = 0.18	1.45 (0.97, 2.17) *p* = 0.07	x	0.69 (0.58, 0.82) *p* < 0.001	1.07 (0.86, 1.33) *p* = 0.55	2.32 (1.35, 3.98) *P* < 0.01	1.25 1.05, 1.48) *p* = 0.01	x

OR = Odds Ratio * odds ratios of association after adjusting for covariates including age, gender, race, employment status, condition of living and marital status

^x^ Data not collected in this wave

^a^ Odds ratios given per 1 SD increase in loneliness or emotional support score. Loneliness and emotional support were each examined in separate models

### Examining the impact of potential effect modifiers on the association between loneliness and emotional support with depression and increased substance use

Using wave 1 data, there was no significant difference in the association between loneliness and emotional support and the occurrence of depression and increased substance use among those living alone compared to those living with others. Similarly, the associations between loneliness and emotional support and the occurrence of depression and increased substance use were not significantly different between young adults and parents of young adults (p-values for interaction > 0.05). Details of the adjusted models are shown in Table 6.

**Table 6:** The effect modification of living condition and respondent type on associations at wave 1 (adjusted)

	Loneliness^a^					Emotional Support^a^				
	**Depression**	**Increase in Substance Use**				**Depression**	**Increase in Substance Use**			
		Smoking tobacco using cigarettes, pipes or cigar	Smoking tobacco using vapes or e-cigarettes	Drinking alcohol	Marijuana and other substances		Smoking tobacco using cigarettes, pipes or cigar	Smoking tobacco using vapes or e-cigarettes	Drinking alcohol	Marijuana and other substances
	Adjusted OR (95% CI)*					Adjusted OR (95% CI)*				
**Living Conditions**										
Living alone (*N* = 97)	3.12 (1.78, 4.88)	2.76 (1.34, 4.99)	1.00 (0.72, 48.39)	1.52 (0.95, 2.01)	1.78 (0.79, 3.81)	0.56 (0.39, 1.32)	0.96 (0.81, 1.15)	1.05 (0.62, 3.45)	1.21 (0.98, 1.42)	0.96 (0.74, 1.25)
Living with others (*N* = 1120)	2.96 (1.37, 4.72)	1.42 (0.78, 3.19)	1.88 (0.61, 47.13)	1.24 (0.65, 1.73)	2.13 (0.70, 3.49)	0.64 (0.45, 1.12)	0.93 (0.82, 1.22)	0.96 (0.51, 3.20)	1.03 (0.87, 1.28)	0.99 (0.87, 1.17)
*(Product terms) #*	*p* = 0.73	*p* = 0.29	*p* = 1.00	*p* = 0.96	*p* = 0.82	*p* = 0.74	*p* = 0.91	*p* = 1.00	*p* = 0.67	*p* = 0.81
**Respondent type**										
Parents (*N* = 688)	2.65 (2.13, 3.30)	1.41 (1.12, 1.77)	1.70 (0.55, 5.20)	1.25 (1.03, 1.52)	1.91 (1.22, 2.94)	0.70 (0.58, 0.85)	0.86 (0.67, 1.11)	0.92 (0.22, 3.87)	1.02 (0.82, 1.27)	0.85 (0.51, 1.42)
Young adults (*N* = 530)	3.12 (2.51, 3.88)	1.84 (1.46, 2.32)	1.73 (0.56, 5.29)	1.17 (0.96, 1.42)	2.56 (1.64, 3.99)	0.66 (0.54, 0.80)	0.83 (0.64, 1.07)	0.77 (0.18, 3.20)	0.93 (0.75, 1.16)	0.83 (0.50, 1.39)
*(Product terms) #*	*p* = 0.33	*p* = 0.14	*p* = 0.98	*p* = 0.67	*p* = 0.28	*p* = 0.63	*p* = 0.82	*p* = 0.81	*p* = 0.56	*p* = 0.93

# p-values of the product terms

* odds ratios of association after adjusting for covariates including age, gender, race, employment status, condition of living and marital status

^a^ Odds ratios given per 1 SD increase in loneliness or emotional support score

### Association between loneliness and emotional support with the occurrence of depression and increase in substance use, adjusting for one and other and the co-variates

Using wave 1 data, loneliness and emotional support were simultaneously included in the same model with other covariates. Loneliness showed a positively significant association with depression and increase in substance use behavior. However, the association between emotional support and the occurrence of depression and substance use became non-significant after including loneliness and other co-variates in the model. Details are shown in Table 7.

In the wave 2 study, loneliness showed a positively significant association with depression, but a non- significant association with increase in substance use after adjusting for emotional support and other co-variates including age, gender, race, employment status, condition of living and marital status. However, emotional support showed a non-significant association with both depression and increase in substance use after adjusting for loneliness and other covariates. Details are shown in Table 7.

**Table 7:** The combined role of the exposure variables on the associations at both waves 1 and 2 (adjusted)

	Wave 1 Adjusted OR (95%CI)					Wave 2 Adjusted OR (95%CI)				
	**Depression**	**Increase in Substance Use**				**Depression**	**Increase in Substance Use**			
		Smoking tobacco using cigarettes, pipes, or cigar	Smoking tobacco using vapes or e-cigarettes	Drinking alcohol	Marijuana and other substances		Smoking tobacco using cigarettes, pipes, or cigar	Smoking tobacco using vapes or e-cigarettes	Drinking alcohol	Marijuana and other substances
Loneliness^a^*	2.45 (2.06, 2.90) *p* < 0.001	1.44 (1.10, 1.88) *p* = 0.01	1.84 (1.06, 3.19) *p* = 0.03	1.34 (1.15, 1.57) *p* < 0.001	1.55 (1.20, 2.01) *p* < 0.001	3.13 (2.47, 3.98) *p* < 0.001	1.36 (0.92, 2.02) *p* = 0.12	1.32 (0.66, 2.64) *p* = 0.43	1.02 (0.72, 1.42) *p* = 0.93	x
Emotional Support^a#^	0.95 (0.80, 1.12) *p* = 0.51	0.85 (0.65, 1.12) *p* = 0.25	1.27 (0.67, 2.40) *p* = 0.47	1.21 (1.02, 1.45) *p* = 0.03	1.01 (0.78, 1.31) *p* = 0.93	0.92 (0.75, 1.13) *p* = 0.43	0.94 (0.64, 1.37) *p* = 0.85	2.71 (0.75, 9.76) *p* = 0.13	1.53 (1.00, 2.34) *p* = 0.05	x

OR = odd ratios

* Adjusted for emotional support and covariates including age, gender, race, employment status, condition of living and marital status

^#^ Adjusted for loneliness and covariates including age, gender, race, employment status, condition of living and marital status

^a^ Odds ratios given per 1 SD increase in loneliness or emotional support score.

^x^ Data not collected in this wave

### The relationship between loneliness or emotional support at wave 1 and the development of depression or increased substance use at wave 2

The results showed a significant positive association between wave1 loneliness and wave 2 depression (OR: 2.34, 95%CI: 1.79–3.04, *P* < 0.001). However, no significant association was found between loneliness and increased substance use parameters.

On the other hand, baseline emotional support demonstrated a significant negative association with follow-up depression (OR: 0.66, 95%CI: 0.55–0.79, *P* < 0.001), whereas emotional support was not significantly associated with substance use behaviour. Details are shown in Table 8.

**Table 8:** Association between wave 1 loneliness or emotional support with wave 2 depression or increased substance use

	Adjusted OR (95%CI)*			
	**Depression** ^**@**^	**Increase in Substance Use** ^**@**^		
		Smoking tobacco using cigarettes, pipes, or cigar	Smoking tobacco using vapes or e-cigarettes	Drinking alcohol
Loneliness^a#^	2.34 (1.79, 3.04) *P* < 0.001	1.13 (0.79, 1.62) *P* = 0.50	1.31 (0.43, 3.96) *p* = 0.64	1.09 (0.83, 1.42) *P* = 0.54
Emotional Support^a#^	0.66 (0.55, 0.79) *p* < 0.001	1.18 (0.66, 2.12) *p* = 0.57	2.12 (0.48, 9.32) *p* = 0.32	1.46 (0.96, 2.22) *p* = 0.08

^a^ Odds ratios given per 1 SD increase in loneliness or emotional support score

# loneliness or emotional support at baseline (wave 1)

@ Depression or increased substance use at follow-up (wave 2)

* odds ratios of association after adjusting for covariates including age, gender, race, employment status, condition of living and marital status

## Discussion

### Main findings

This current cohort study used the information of recruited participants from three ongoing cohort studies in the metropolitan New York City area to determine the association of loneliness and emotional support with the occurrence of depression and substance use behavior among young adults and their parents during the COVID-19 pandemic. Findings from this study showed that individuals who experience higher levels of loneliness were more likely to experience moderate to severe depressive symptoms and an increase in substance use behavior compared to those who experience lower levels of loneliness. Also, those who experienced a high level of emotional support were less likely to experience moderate to severe depression and an increase in substance use behavior compared to those who had low level of emotional support. In addition, the associations were generally stronger in young adults compared to their parents, but the differences were non-significant. There was also no significant difference in these associations based on living alone or living with others.

### Loneliness, emotional support and mental health outcomes

This study reported the prevalence of moderate to severe depressive symptoms among participants in wave 1 to be 18.23%. This is similar to the prevalence reported in some other studies conducted during the COVID-19 pandemic [15, 16]. Lei and colleagues recorded a prevalence of 14.6% of depression among adults from southwestern China [16]. A body of literature reported a pooled prevalence of depression of 25% (95% CI: 18 − 33%) of 12 studies that were conducted during the COVID-19 pandemic among adults from China, Vietnam, India and Europe [15]. The literature compared this to a global estimated prevalence of depression of 3.44% in 2017 which was before the pandemic [15]. This further helps to understand the impact of the COVID-19 pandemic on mental health outcomes.

This study recorded a very high prevalence (67.82%) of alcohol use among participants and an increase in alcohol consumption during the pandemic. This finding is consistent with some previous studies [17–20]. For example, Czeisler and colleagues reported a 40.9% prevalence of alcohol consumption among U.S. adults and that 13.3% of these participants initiated or increased substance use as a coping strategy during the COVID-19 pandemic [18]. However, there is a disparity in those findings compared to another study that found a decrease in alcohol consumption. Sallie and colleagues documented a significant decrease in alcohol consumption among adults from 85 different countries when comparing the pre-quarantine to the quarantine period [21]. They attributed the decrease in alcohol consumption during the quarantine period to decrease in availability of alcohol to the reach of immediate households and reduction to alcohol cues and social contexts that lead to the urge to drink [21].

A significant positive association between loneliness and the occurrence of depressive symptoms was observed in this study. This finding is not so different from some other studies [22–24]. For example, Groarke and colleagues found that loneliness is positively associated with depressive symptoms and that depressive symptoms also predicts higher levels of loneliness after one month [23]. Hoffart and colleagues also had similar findings of a significant positive association between loneliness with depressive symptoms and anxiety but reported a stronger association with depressive symptoms [24]. There was also a significantly positive association found between loneliness and increased substance use in this study. The finding is consistent with other studies and further explains that loneliness increases the risk of substance use [25, 26]. However, Segrin and colleagues showed that the association found between loneliness and substance use were not prospective direct effects but a significant indirect effect which was as a result of increased stress [26]. Similarly, a study among child and adolescent substance users reported a significant positive correlation between loneliness and substance use proclivity scale [27].

In this current study, emotional support has been found to be negatively associated with depression. This is largely consistent with findings from other studies [28, 29]. However, most of those studies reported findings for social support instead of emotional support as social support encompasses emotional support, as well as other forms of support. A study among adults experiencing self-isolation during the COVID-19 pandemic reported that higher levels of social support is associated with 63% lower risk for elevated depressive symptoms and 52% lower risk of poor sleep quality compared to low levels of support [28]. However, our study reported a non-significant association between emotional support and increase in substance use behavior indicators except for increase in smoking tobacco using cigarettes, pipes, or cigar. This finding is in contrast with the findings of other studies [30, 31]. A study found that increase in general support is associated with reduction in drug use severity and that increased peer support involving mutual help was associated with large improvement in alcohol use severity [30]. Similarly, a cross-sectional study among 1,598 students documented that students with higher perceived social support reported less alcohol consumption compared to students with lower level of support during the COVID-19 pandemic [31]. This may be because individuals with high social support systems find it easier to cope with the distress of the pandemic than others. Some studies have found that social support has a moderating role in the associations involving substance use and depression rather than a direct relationship to these mental health outcomes [32, 33]. This is related to our findings when loneliness and emotional support was included in the same model with other covariates, the associations were consistent with a model whereby loneliness mediated the association between emotional support and the mental health outcomes; depression and increase in substance use. Therefore, it is important that interventions focused on increasing emotional support to improve mental health outcomes should be designed in such a way that loneliness is also addressed.

### Effect modification by respondent types and living conditions

This study found that estimates of association for poor mental health outcomes associated with loneliness or lower emotional support were numerically stronger for young adults compared to older adults. However, the modifier effect of age was not statistically significant. Young adults may likely be prone to poor mental health outcomes during lockdowns as a result of factors including reduced income, loss of employment, increased exposure to social media and news relating to COVID-19 and reduced access to mental health care service due to the pandemic. Studies have shown that age influences mental health outcomes, but the association remains unclear. Some studies have reported that older age is associated with lower risk of mental health problems [18, 34]. A study among US adults reported that participants who were 65 years and older reported a lower percentage of depressive disorder (5.8%) and other mental health problems compared to those of younger age [18].

The modifier effect of living condition in the association between loneliness and emotional support with the occurrence of depression and substance use behavior was found to be non-significant. Therefore, our study suggests that there is no significant difference in these associations in those living alone compared to those living with others. Some other studies considered the direct link between living conditions and mental health outcomes [16, 35, 36]. A longitudinal study of youth in Canada reported that there is a direct relationship between living alone and higher risk of mood symptoms and substance use compared with living with two or more people and with family members [35]. Similarly, Sylvestre and colleagues found a direct association between living alone and higher risk of substance use disorders during the COVID-19 pandemic [36]. Some studies also classified living conditions differently. For example, Hawke and colleagues classified living conditions into; those living in suburban or urban communities and large households, and those living in rural communities and smaller households respectively. They found that youth who were living in urban or suburban areas, in larger households were mostly vulnerable to mental health problems during the pandemic compared to those living in smaller cities, town, villages, and rural areas, in smaller households, especially those with poor mental and physical health baseline [35]. Another study that classified living condition in terms of household income reported that lower income households had higher scores on the self-rating anxiety scale (SAS) and the self-rating depression scale (SDS) compared to others [16].

### Strengths and limitations of the study

In this study, unlike in some of the other studies, we used standard/validated scales for depression, loneliness and emotional support. In addition, the evaluation of potential confounding effects and effect modification in this study helped to further provide better understanding of studied associations.

However, there were some missing data at wave 2, which is common with follow-up studies. Information about marijuana and other drug use was not available at wave 2. Therefore, differences in wave 1 versus wave 2 findings could be due, in part, to participant differences. However, we did adjust for basic demographic characteristics in each wave. Although the study adjusted for some covariates as potential confounders, there is still the possibility of residual confounding effects of some other unmeasured factors.

The study focused on a cross-sectional analysis of the data, allowing comparison across the two COVID-19 waves, but leaving the potential of reverse causation. However, prospective analyses confirmed the associations of wave 1 loneliness and emotional support with wave 2 depression, whereas the prospective associations were not statistically significant for increased substance use.

Some participants in wave 2, who already increased their substance use in wave 1 and were less likely to further increase use in wave 2 might not be suitable references. However, reported increased substance use in wave 2 may help understand how the progression of the pandemic affected substance use behavior. Additionally, the participants’ information used in this study was self-reported and subjective, which increases the possibility of some potential bias. However, self-reported studies are common in such research as is the use of validated scales/tools to collect the information.

Despite these limitations, this study contributes greatly to existing knowledge about the association between loneliness and emotional support, especially with the occurrence of depression and substance use behavior in the context of the COVID-19 pandemic. Consequently, the findings presented here could be useful in designing interventions targeting mental health outcomes in future pandemics and similar situations.

## Conclusions

This study demonstrates that higher loneliness levels were associated with increased likelihood of the occurrence of depression and increase in substance use behavior among adults during the COVID-19 pandemic, while greater emotional support was associated with reduced likelihood of the occurrence of depression. However, emotional support did not significantly reduce the likelihood of increased substance use behavior in adults. In addition, these relationships did not differ based on living alone compared to those living with others, or among young adults compared to their parents. Therefore, interventions designed to provide emotional support and the reduction of loneliness would be useful for improving mental health outcomes in future pandemics.

The U. S. Surgeon General Dr. Vivek Murthy has highlighted the contribution of loneliness and isolation to mental health challenges in the United States [37]. To address the negative consequences of loneliness and isolation, he laid out in a framework consisting of six fundamental pillars including strengthening social infrastructure, enacting pro-connection public policies, mobilizing the health sector, reforming digital environment, deepening knowledge and cultivating a culture of connection. The framework is designed as a national strategy to promote social connection and improve mental health outcomes [37].

## Data Availability

The data used for this study is not publicly available due to ongoing analyses but can be requested from Christina W. Hoven.

## Abbreviations

COVID-19: Coronavirus Disease-2019
GPEG: Global Psychiatric Epidemiology Group
NIH: National Institute of Health
NYC: New York City
NYSPI: New York State Psychiatric Institute
PTSD: Post-Traumatic Stress Disorder
U.S.: United States
WHO: World Health Organization
